# The Fight Just Born—Neonatal Cancer: Rare Occurrence with a Favorable Outcome but Challenging Management

**DOI:** 10.3390/cancers14092244

**Published:** 2022-04-29

**Authors:** Maria Antonietta De Ioris, Francesco Fabozzi, Mariachiara Lodi, Giulia Vitali, Maria Debora De Pasquale, Giada Del Baldo, Rachid Abbas, Emanuele Agolini, Alessandro Crocoli, Chiara Iacusso, Giuseppe Maria Milano, Annalisa Serra, Angela Mastronuzzi

**Affiliations:** 1 Department of Hematology/Oncology, Cell and Gene Therapy, Bambino Gesù Children’s Hospital, IRCCS, 00165 Rome, Italy; francesco.fabozzi@opbg.net (F.F.); mariachiara.lodi@opbg.net (M.L.); mdebora.depasquale@opbg.net (M.D.D.P.); giada.delbaldo@opbg.net (G.D.B.); giuseppemaria.milano@opbg.net (G.M.M.); annalisa.serra@opbg.net (A.S.); angela.mastronuzzi@opbg.net (A.M.); 2 Department of Pediatrics, University of Tor Vergata, 00165 Rome, Italy; 3 Department of Maternal Infantile and Urological Sciences, Sapienza University of Rome, 00165 Rome, Italy; giulia.vitali@uniroma1.it; 4 INSERM, Centre for Research in Epidemiology and Population Health (CESP), U1018 Villejuif, France; abbas.rachid@gmail.com; 5 Laboratory of Medical Genetics, Bambino Gesù Children’s Hospital, IRCCS, 00165 Rome, Italy; emanuele.agolini@opbg.net; 6 Surgery Department, Bambino Gesù Children’s Hospital, IRCCS, 00165 Rome, Italy; alessandro.crocoli@opbg.net (A.C.); chiara.iacusso@opbg.net (C.I.)

**Keywords:** cancer, children, newborn, cancer predisposition syndrome

## Abstract

**Simple Summary:**

Neonatal cancer represents a heterogeneous group of neoplasms with a wide range of clinical, biological, and prognostic features. Characterizing genetic cancer risk is critical for improving short- and long-term patient care, notably in this category of patients. In this article we aimed to describe the main features of neonates diagnosed with cancer in our centre during a 15-year period and to emphasize the importance of genetic screening and its implication in treatment strategies and prognosis.

**Abstract:**

The occurrence of cancer in newborns within the first 28 days of life is uncommon, with different clinical presentation from other age groups. Prenatal diagnosis is reported in about half of patients, while a genetic predisposition condition is supposed. The management of a newborn with cancer can be challenging and needs to be tailored according to the histology and the primary tumor site; surgery represents the main strategy, while chemotherapy should be considered with caution because of the higher toxicity and mortality due to different pharmacokinetics in neonates compared to older children. We describe the first Italian series over a 15-year period of patients affected by both benign and malignant neoplastic diseases diagnosed within the first 28 days of life; 74 newborns were diagnosed with neonatal tumors, representing 1.5% of the cancer population in the same period, and a prevalence of germ cell tumors (55%) and neuroblastoma (16%) was observed. Surgery was performed on 80% of patients, while chemotherapy was necessary for about 20% of patients. The 5-year overall survival (OS) exceeded 90%; treatment-related deaths are a major concern, representing 80% of overall deaths. A genetic/syndromic condition was detected in 16% of the population; additionally, a cancer predisposition syndrome (CPS) was identified in about 10% of patients. According to our experience, all newborns affected by cancer should warrant genetic counselling and a screening test for CPS.

## 1. Introduction

The occurrence of cancer in newborns within the first 28 days of life is uncommon. According to the Surveillance Epidemiology and End Results (SEER) report from the National Cancer Institute, the incidence of neonatal tumors is predicted to be 1 per 27,000 to 33,300 live births, with a mortality rate of 1 per 6 to 24 million live births [1]. Neonatal cancer characteristics, including the clinical presentation, the tumor type, the biological and histological features, and the response to treatment significantly differ from other pediatric tumors. Prenatal diagnosis is reported in about half of patients, resulting in early management that could improve the outcome [2]. Considering the low occurrence rate, the origin of neonatal tumors is still unclear, and the genetic landscape of these tumors is not well known [3,4], but due to its early onset, it has been proposed that genetic factors could promote their development. Less than 10% of childhood malignancies are linked to a well-known cancer predisposition syndrome (CPS), such as *RB1* mutation in retinoblastoma or *TP53* mutations in brain tumor, bone or soft-tissue sarcoma and adrenocortical carcinoma [5]. The identification of a genetic cancer risk is critical for planning treatment, arranging genetic counseling and ensuring adequate follow-up [6]. The management of a newborn with cancer can be challenging and needs to be tailored according to the histology and the primary tumor site; surgery represents the main strategy in most cases, while chemotherapy should be considered with caution. The drug distribution, metabolism and excretion differ qualitatively and quantitatively in neonates compared to older children, resulting in a higher toxicity and a higher mortality rate [4].

Herein, we describe the first Italian case series of patients affected by neoplastic disease diagnosed within the first 28 days of life and referred to the Bambino Gesù Children’s Hospital in Rome over a 15-year period.

## 2. Materials and Methods

Data were obtained through a retrospective chart review of patients admitted at Bambino Gesù Children’s Hospital from 1 January 2006 to 31 December 2021. All children histologically diagnosed with both benign and malignant tumors within 28 days of life were included. The medical records of patients were reviewed and data regarding gender, age, prematurity, pregnancy history, tumor localization at diagnosis, possible presence and site of metastases, imaging findings, histology, association with other disease or dysmorphic features, treatment plan (surgery and/or chemotherapy), and outcome were collected. The first clinical and/or radiological evidence of malignancy was considered as the date of diagnosis. Patient data were analyzed using IBM SPSS Statistics Version 22 for Windows. The Kaplan–Meier method was used to estimate overall survival (OS) and event-free survival (EFS). The OS was defined as the time from diagnosis to death, whatever the cause. The EFS was defined as the time from diagnosis to relapse, progression or death, whatever the cause. This retrospective study was approved by the Internal Review Board (IRB). All investigations were conducted according to principles expressed in the Declaration of Helsinki, and all parents of patients signed an informed consent before performing the genetic analysis and before giving any treatment.

## 3. Results

**Figure 1:**
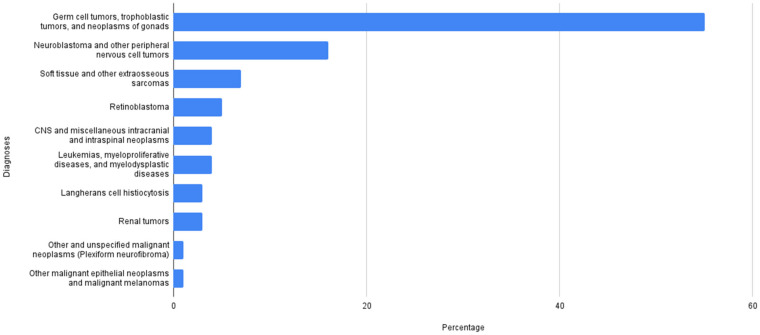
Histology distribution of the studied population (*N* = 74).

In the 15-year period from 2006 to 2021, 74 consecutive patients within 28 days of life were diagnosed with neonatal tumors. They represent 1.5% of the cancer population in the same period. The most common histology is germ cell tumor (GCT) (55%), followed by neuroblastoma (16%) and soft-tissue sarcomas (7%) (for details see Figure 1).

Patients’ characteristics are summarized in Table 1. The sex ratio and the histology distribution differ from older populations. There is a marked excess of females in our population, with an overall ratio of 1.85:1. This is probably due to the high percentage of females among babies diagnosed with GCT, accounting for 75% of them. On the other hand, neuroblastoma is slightly more common among males, with a ratio of 1.4:1 in our cohort.

Prenatal diagnosis was performed in 20 out of 72 cases (27%): 15 were GCTs (37% of all GCTs), two central nervous system (CNS) tumors, one adrenal carcinoma, one fibrosarcoma, and one neuroblastoma. The number of premature babies (born before 36 weeks of gestational age) was five, representing 7% of the population. Six out of seventy-four patients (8%) presented a CPS, while six patients presented a malformative/genetic condition (see Table 2 for details).

Overall, our cohort includes 23 neonates with benign or low-malignancy tumors, namely both mature and immature teratoma, congenital mesoblastic nephroma, infantile fibrosarcoma, myofibroma, ganglioglioma, and plexiform neurofibroma.

Metastases were detected in 9 out of 74 patients.

Fifty-eight of the seventy-four (80%) infants underwent successful surgery, while two patients had severe infectious complications after surgery, leading to their death.

Twenty of the seventy-four patients (27%) received carboplatin-based chemotherapy; two infants died from infectious complications occurring during the neutropenia phase associated with therapy-related organ toxicity leading to multi-organ failure. Similarly, two patients had severe infectious complications after tumor biopsy, leading to their death. Only one patient died from progressive disease.

The median follow-up time was 4.7 years (range 0–178 months, IQR 2–7.3 years). At last follow-up, five children had died (7%): two with soft-tissue sarcoma (an alveolar rhabdomyosarcoma and an extra-renal rhabdoid tumor), one with neuroblastoma, and one with acute myeloid leukemia (AML) and later affected by CNS GCT. The median time from diagnosis to death was 61 days; the disease progression was the cause of death in one metastatic rhabdomyosarcoma, while the treatment was the cause of death in four patients as previously discussed. Out of nine metastatic tumors, two patients died. Only one death occurred (4%) among neonates with benign or low-malignancy tumors, whereas 4 out of 51 (8%) newborns affected by malignant tumors died. The estimated 5-year OS was 92% (95% CI, 83.8–96.2), while the 5-year EFS was 86.2 (95% CI, 74.4–93.1).

### 3.1. Extracranial GCTs

Extracranial GCTs represent the most common tumors in our cohort, diagnosed in 40 out of 74 children (54%). Teratomas are the most common, accounting for 45% of the cases, followed by germinomas (30% of the cases) and finally yolk sac tumors, occurring in 25%. The primary site was in the coccygeal region (82%). Five GCTs were in the head and neck region (12%), whereas only one was cardiac, and one intra-abdominal. All children were treated by surgery without the need for chemotherapy. The characteristics of all patients with extracranial GCT are summarized in Table S1.

### 3.2. Neuroblastoma

Neuroblastoma was diagnosed in 12 children, accounting for the 16% of all diagnoses. Five neuroblastomas were localized according to the International Neuroblastoma Risk Group Staging System (INRGSS): three patients L1 and two patients L2. Seven patients were metastatic: six presented a metastatic special (Ms) stage and one later presented a metastatic (M) stage. *MYCN* gene amplification was detected in one patient, while segmental chromosomal abnormalities (SCA) were reported in two later patients. Six patients needed chemotherapy, with chemotherapy in addition to surgery in three of them, while surgery alone was performed on four patients. After biopsy, an observational approach was followed in two patients. Overall, only one patient died from infectious complications. The characteristics of all patients with neuroblastoma are summarized in Table S2.

### 3.3. Soft-Tissue Sarcomas

Five children were diagnosed with soft-tissue sarcomas: two rhabdomyosarcomas (one alveolar and one embryonal), a malignant rhabdoid tumor (MRT) arising in the head/neck region, a congenital fibrosarcoma and a myofibroma. Only the alveolar rhabdomyosarcoma was metastatic. The extra-renal rhabdoid tumor did not carry *SMARCA4* or *SMARCB1* mutations. Surgery was performed on all patients, while chemotherapy was administered in three patients. Overall, two children affected by rhabdoid tumor and alveolar rhabdomyosarcoma died. The characteristics of all patients with soft-tissue sarcoma are summarized in Table S3.

### 3.4. Retinoblastoma

Among our cohort, three cases presented a positive family history and carried a hereditary *RB1* germline mutation. No patient underwent enucleation since all were successfully treated with chemotherapy alone. The characteristics of all patients affected by retinoblastoma are summarized in Table S4.

### 3.5. CNS Tumors

Four cases of CNS tumors were diagnosed: a ganglioglioma, a choroid plexus carcinoma, a CNS GCT, and an infantile high-grade glioma with classical *ETV6/NTRK3* fusion. All neonates underwent surgery and two were also treated with chemotherapy. All children are alive. In this group, only the baby affected by a large CNS mature teratoma died in the sixth day of life, two days after biopsy. The characteristics of all patients affected by CNS tumors are summarized in Table S5.

### 3.6. Hematologic Malignancies

Three patients were affected by acute leukemia: myeloid in two and lymphoblastic in the latter one. A treatment-related death occurred in a patient affected by AML. Of note, cases of transient abnormal myelopoiesis that can occur in Down syndrome or Noonan syndrome were not included in our cohort. The characteristics of patients affected by leukemia are included in Table S6.

### 3.7. Renal Tumors

Two mesoblastic nephromas (classic and cellular subtype) were identified, treated by surgery alone successfully. The characteristics of patients affected by mesoblastic nephromas are included in Table S6.

### 3.8. Others

Langerhans cell histiocytosis (LCH), an adrenocortical carcinoma associated with Li-Fraumeni syndrome, and a plexiform neurofibroma associated with neurofibromatosis type 1 (NF1) completed the series. All children are alive. The characteristics of these patients are included in Table S6.

## *4.* Discussion

Cancer is a rare occurrence in newborns accounting for about 2% of all pediatric tumors. Neonatal cancers are heterogeneous with respect to the histological point of view and their behavior, and the oncological data available on this specific population are limited [1,2,7,8].

In this paper, we describe a unique cohort of 74 neonatal tumors diagnosed before 28 days of life and admitted to our tertiary care pediatric hospital over a 15-year study period, accounting for 1.5% of all childhood tumors diagnosed during the same period in our institution. Out cohort represents the first Italian series as well as one of the largest series reported by a single center. Moreover, it is one of the few cohorts detailing this specific population.

In our population we achieved a more favorable outcome in terms of OS that exceeded 90% at 5 years, higher than the best 5-year OS achieved in the literature reviewed of around 70–85% [2,9]. Moreover, in our series, only one patient died due to progressive disease, while the latter four patients died due to treatment toxicity and infective complications after surgery or chemotherapy. Of note, the deaths are reported among patients who received treatment, namely chemotherapy (2 out of 20, or 10%) and/or surgery (2 out of 58, or 3.5%). Only one patient with metastatic rhabdomyosarcoma died due to progressive disease. We have already described two cases of congenital rhabdomyosarcoma emphasizing that the disease management is often challenging, and especially for the alveolar subtype, the outcome is dismal despite intensified multimodality therapy [10]. In fact, it characteristically manifests with multiple subcutaneous nodules and progression most commonly occurs in the CNS [11]. In this context, we proposed that CNS prophylaxis could play a role in preventing leptomeningeal dissemination, and molecular studies can allow a deeper tumor characterization, treatment stratification and identification of new potential therapeutic targets.

According to our experience, the dismal prognosis is associated with treatment more than with an aggressive disease. Nevertheless, the acute drug toxicities, as the acute surgical complications, do not represent a major concern in an experienced center. The management of a newborn with cancer is critical, accounting as a non-negligible risk of death due to the treatment and should be considered with caution. Indeed, a challenging issue is the drug dosage, considering that the hepatic and renal functions are still immature. In particular, phase 2 hepatic enzymes responsible for acetylation and glucuronidation, as well as the glomerular filtration rate, mature slowly, and reach 50% of the adult value at 48 weeks postmenstrual age [12,13]. Dosage is usually calculated based on body weight rather than body surface area due to errors in length measurement and differences in the percentage of body surface area. According to our experience and considering the pharmacokinetic studies available in this age group, carboplatin seems to be the safest drug [14]. Few pharmacokinetic data about anticancer drugs administered in infants are available, and drug monitoring should be considered [4]. Moreover, the chemotherapy should be administered in experienced centers resulting in limited toxic death, while close monitoring for infection is needed. The infective complication seems to be the major concern in the pediatric population with cancer who underwent surgery or who received chemotherapy. As sepsis is a major concern in the neonatal population resulting in morbidity and mortality [15], infective complications seem to be the major concern in the pediatric population with cancer, representing the cause of death in 80% of cases. Of note again, all deaths occurred in patients who received chemotherapy or/and underwent surgery; the neuroblastoma infant paradigm that reserves treatment only in patients with symptoms related to the tumor or in selected cases with aggressive biology should be considered in all histology in order to avoid treatment-related deaths.

In this setting, it is of utmost importance to improve the molecular characterization, in order to identify new targets for the identification of new biology-driven therapeutic approaches that present a manageable toxicity and reduce secondary incidence of neutropenia, such as vemurafenib, a *BRAF* kinase inhibitor we used in the treatment of LCH with negligible adverse effects [16]. A more impressive paradigmatic situation is represented by infantile high-grade gliomas. Recent discoveries pointed out that these neoplasms, with a clinical course that differs from their pediatric and adult counterparts, carry specific molecular alterations, namely kinase (*ALK*, *NTRK1/2/3*, *ROS1*, or *MET*) gene fusions [17]. Significant research has evaluated the underlying molecular genetic make-up of pediatric brain tumors, with the most recent World Health Organization (WHO) classification putting more emphasis on molecular characteristics and removing some previously used nomenclature based on histologic architecture alone [18]. In the context of a major risk of treatment-related toxicity, the possibility to target these gene fusions with different “intelligent” drugs may play a role in this fragile population [19].

The neonatal population has a high rate of accompanied congenital anomalies and cancer predisposition syndromes. Considering the early tumor onset, it has been proposed that genetic factors could promote their development and CPS may be suspected in this population [5]. In this series, 8% presented a well-known CPS, overlapping with the 10% reported in the whole pediatric cancer population. Moreover, another 8% present a syndromic condition resulting in a higher prevalence of genetic/syndromic conditions in this group, as observed [4,5]. The recognition of CPS with or without associated congenital anomalies is crucial to improve diagnosis and clinical management. Further analyses are needed to clarify the genetic landscape in this age group; of note, even the sporadic retinoblastoma patient presented a *RB1* germline mutation. The improvement at a lower cost of next-generation sequencing technologies is likely to improve the understanding of the complex landscape in the near future [20]. Recently, Yeh et al. proposed a model-based approach to estimate the clinical impact of universal genetic screening in newborns for pediatric CPS [21]. Their findings suggest that under the best-case assumption of full adherence to screening and surveillance guidelines, targeted next-generation sequencing (t-NGS) would identify approximately 1580 individuals with pathogenic or likely pathogenic variants among 3.7 million newborns each year in the US. The identification of a genetic condition would allow the modulation of treatment according to the oncological risk, and the genetic test should be extended to the whole family with genetic counseling [5]. Therefore, we recommend genetic counseling and possible germline genetic testing in all patients diagnosed with pediatric cancer in the first month of life.

Finally, a prenatal diagnosis was obtained in about 20% of cases that should be discussed/referred to a pediatric hospital with oncology and surgery dedicated services. The pregnancy should be monitored by a multidisciplinary team including gynecologists, obstetricians, neonatologists, oncologists and radiologists experienced in neonatal diagnostics, and neonatal surgeons [4,22]. The timing and method of delivery should be tailored, taking prematurity and estimated birthweight into account, as well as the tumor volume and the rupture risk. A preterm delivery may be considered in case of risk of maternal or fetal death, or when early treatment is needed with an impact on prognosis [4,23]. Infants represent a population with a high sensitivity to ionizing radiation damage [24]. A diagnostic tool should be chosen with attention, and MRI and ultrasound should be preferred. For the same reason, the use of radiotherapy treatments is not acceptable in this age group and should be omitted [25]. Considering the clinical implication in terms of mortality of treatment and the importance of genetic counseling, a newborn with cancer should be addressed to an experienced center, before delivery if possible, to receive the most appropriate diagnostic and therapeutic approach.

## 5. Conclusions

This series represents the first and largest Italian case series of patients with cancer in the neonatal period. Although it is a retrospective evaluation, it provides many insights for prospective studies.

Neonatal tumors represent a rare occurrence: less than 2% of the pediatric cancer population. Above all, the outcome is favorable with an excellent OS survival that exceeded 90%, while relapse or progression is rare. The management should be considered with caution and in experienced centers considering the impressive 5% mortality rate related to treatment; in our experience, the treatment-related mortality rate is mainly due to infective complications. In the general neonatal population, sepsis represents an important cause of morbidity and mortality rate, which should be considered. Considering the favorable outcome and the consistent mortality rate related to treatment, which should be considered with caution, chemotherapy should be reserved for patients who actually need this treatment. The neuroblastoma infant approach that reserves treatment only in patients with symptoms related to the tumor or in selected cases with aggressive biology should be considered in all histology in order to avoid treatment-related deaths. Nevertheless, chemotherapy is manageable in experienced centers with the major concern of severe infection during neutropenia. Moreover, infective complication is the main issue in the post-operative period accounting of major complications and deaths. In this setting, target therapy needs to be considered and a major effort should be addressed to identify possible target treatments in order to avoid chemotherapy.

Neonatal tumors seem to present a complex genetic landscape considering both CPS and syndromic condition; extensive genetic studies should be addressed in all cases in order to achieve better management in terms of treatment and family counseling. Further genetic information will clarify the pathophysiology of neonatal cancers. According to our experience, genetic counselling and the CPS screening should be addressed to all newborns with cancer.

## Figures and Tables

**Table 1:** Patients’ characteristics in the studied population (N = 74).

Sex Ratio (M/F)	26/48
**Median Age at diagnosis**	11 days (1–28 days)
**Prenatal diagnosis**	20 (27%)
**Prematurity**	5 (7%)
**Surgery**	58 (78%)
**Chemotherapy**	20 (27%)
**CPS**	6 (8%)
**Congenital Defects**	6 (8%)

**Table 2:** This table resumes the characteristics of children with Cancer Predisposition Syndrome or congenital defects.

Pt	Sex	Age(d)	Diagnosis	Metastasis	Prenatal Diagnosis	Prematurity	Outcome	Genetic Study	Congenital Defects
1	M	11	Retinoblastoma	NO	NO	NO	Alive	*RB1* germline mutation	
2	F	3	Neuroblastoma	YES, liver	NO	NO	Alive	Waardenburg Syndrome	Atrial sept defect, Aortic Coarctation, esophageal atresia
3	M	20	Retinoblastoma	NO	NO	NO	Alive	*RB1* germline mutation	
4	M	6	Plexiform neurofibroma	NO	NO	YES	Alive	*NF1*	
5	M	0	Retinoblastoma	NO	NO	NO	Alive	*RB1* germline mutation	.
6	F	1	Adrenal Cortical carcinoma	NO	YES	NO	Alive	Li-Fraumeni Syndrome	
7	F	5	Glioblastoma	NO	YES	NO	Alive	Negative	Sensorineural hearing loss
8	F	18	Germ Cell Tumors	NO	YES	NO	Alive	Negative	Sensorineural hearing loss
9	F	13	Neuroblastoma	NO	NO	YES	Alive	Not performed	Atrial septal defect, congenital single kidney
10	M	3	Leukemia	NO	NO	NO	Died	Not performed	Fallot tetralogy
11	F	1	Germ Cell Tumors	NO	YES	YES	Alive	Not performed	Congenital urethra atresia, ureterovaginal fistula
12	M	10	Germ Cell Tumors	NO	NO	NO	Alive	Not performed	Intestinal duplication

## Supplementary Materials

The following supporting information can be downloaded at: https://www.mdpi.com/article/10.3390/cancers14092244/s1, Table S1: Clinical characteristics of newborns with extracranial GCT are summarized; Table S2: Clinical characteristics of newborns affected by neuroblastoma are summarized; Table S3: Clinical characteristics of newborns affected by soft tissue sarcoma are summarized; Table S4: Clinical characteristics of newborns with retinoblastoma are summarized. Table S5: Clinical characteristics of newborns affected by CNS tumors are summarized; Table S6: Clinical characteristics of newborns affected by other malignancies are summarized.
