# Supplementary material for: The Fight Just Born—Neonatal Cancer: Rare Occurrence with a Favorable Outcome but Challenging Management
Source: Cancers (Basel). 2022 Apr 29;14(9):2244. doi: 10.3390/cancers14092244 (PMC9103742; doi:10.3390/cancers14092244)
Supplement: Supplementary file 1 [file cancers-14-02244-s001.zip › cancers-1680174-supplementary.pdf]

**Table S1.** Clinical characteristics of newborns with extracranial GCT are summarized. NA = not applicable.

| Sex | Age (Days) | Diagnosis | Localization | Metastasis | Prenatal Diagnosis | Chemotherapy | Surgery | Relapse | Outcome |
|---|---|---|---|---|---|---|---|---|---|
| F | 2 | Malignant Teratoma | sacrococcygeal | no | no | no | yes | no | alive |
| F | 7 | Germinoma | sacrococcygeal | no | no | no | yes | no | alive |
| F | 0 | Germinoma | sacrococcygeal | no | no | no | yes | no | alive |
| F | 0 | Malignant Teratoma | sacrococcygeal | no | no | no | yes | no | alive |
| M | 5 | Yolk sac tumor | sacrococcygeal | no | no | no | yes | no | alive |
| F | 5 | Yolk sac tumor | sacrococcygeal | no | yes | no | yes | no | alive |
| M | 0 | Yolk sac tumor | heart | no | yes | no | yes | no | alive |
| F | 3 | Mature teratoma | sacrococcygeal | no | yes | no | yes | no | alive |
| M | 16 | Germinoma | sacrococcygeal | no | no | no | yes | no | alive |
| F | 5 | Germinoma | head/neck region | no | yes | no | yes | no | alive |
| F | 21 | Yolk sac tumor | sacrococcygeal | no | no | no | yes | no | alive |
| F | 4 | Germinoma | sacrococcygeal | no | no | no | yes | no | alive |
| F | 1 | Germinoma | sacrococcygeal | no | yes | no | yes | no | alive |
| F | 3 | Germinoma | sacrococcygeal | no | no | no | yes | no | alive |
| M | 23 | Germinoma | sacrococcygeal | no | no | no | yes | no | alive |

| | | | | | | | | | |
|---|---|---|---|---|---|---|---|---|---|
| F | 21 | Yolk sac tumor | head/neck region | no | yes | no | yes | no | alive |
| F | 20 | Germinoma | sacrococcygeal | no | yes | no | yes | no | alive |
| F | 15 | Germinoma | sacrococcygeal | no | no | no | yes | no | alive |
| M | 15 | Germinoma | sacrococcygeal | no | no | no | yes | yes | alive |
| F | 19 | Germinoma | sacrococcygeal | no | no | no | yes | no | alive |
| F | 0 | Immature teratoma | sacrococcygeal | no | no | no | yes | no | alive |
| F | 17 | Immature teratoma | sacrococcygeal | no | no | no | yes | no | alive |
| F | 0 | Mature teratoma | sacrococcygeal | no | yes | no | yes | no | alive |
| F | 16 | Immature teratoma | sacrococcygeal | no | no | no | yes | no | alive |
| M | 0 | Yolk sac tumor | sacrococcygeal | no | yes | no | yes | no | alive |
| F | 9 | Immature teratoma | sacrococcygeal | no | no | no | yes | no | alive |
| F | 19 | Immature teratoma | sacrococcygeal | no | no | no | yes | no | alive |
| F | 18 | Immature teratoma | head/neck region | no | yes | no | yes | no | alive |
| F | 10 | Immature teratoma | abdomen | no | no | no | yes | Yes, abdomen | alive |
| F | 25 | Yolk sac tumor | sacrococcygeal | no | no | no | yes | no | alive |
| F | 11 | Mature teratoma | sacrococcygeal | no | yes | no | yes | no | alive |

| | | | | | | | | | |
|---|---|---|---|---|---|---|---|---|---|
| F | 18 | Yolk sac tumor | sacrococcygeal | no | yes | no | yes | no | alive |
| F | 27 | Mature teratoma | head/neck region | no | no | no | yes | no | alive |
| F | 17 | Mature teratoma | sacrococcygeal | no | no | no | yes | no | alive |
| M | 15 | Mature teratoma | sacrococcygeal | no | no | no | yes | no | alive |
| F | 13 | Mature teratoma | sacrococcygeal | no | no | no | yes | no | alive |
| F | 14 | Mature teratoma | sacrococcygeal | no | no | no | yes | no | alive |
| M | 15 | Mature teratoma | sacrococcygeal | no | yes | no | yes | no | alive |
| M | 10 | Yolk sac tumor | sacrococcygeal | no | no | no | yes | no | alive |
| M | 13 | Yolk sac tumor | head/neck region | Yes, lymph node | yes | no | yes | no | alive |

**Table S2.** Clinical characteristics of newborns affected by neuroblastoma are summarized. SCA = segmental chromosomal alterations. NA = not applicable. TRM = treatment related mortality

| Sex | Age at Diagnosis (Days) | Stage | Genomic Risk Factors | Localization | Metastasis Site | Prenatal Diagnosis | Chemotherapy | Surgery | Relapse | Outcome | Death Causes |
|---|---|---|---|---|---|---|---|---|---|---|---|
| **F** | 11 | Ms | NA | adrenal | bone | no | no | yes | no | alive | NA |
| **F** | 13 | L1 | NA | adrenal | NA | no | yes | yes | no | alive | NA |
| **F** | 3 | Ms | NA | adrenal | liver | no | no | no | no | alive | NA |
| **F** | 6 | Ms | NA | paravertebral | liver | no | yes | no | yes | alive | NA |
| **M** | 17 | L1 | NA | adrenal | NA | yes | no | yes | no | alive | NA |
| **M** | 22 | Ms | SCA | adrenal | bone, bone marrow, liver, skin, paraspinal | no | yes | yes | no | alive | NA |
| **M** | 1 | Ms | SCA | pelvic | liver | no | no | no | no | alive | NA |
| **M** | 17 | L1 | NA | adrenal | NA | no | no | yes | no | alive | NA |
| **M** | 1 | L2 | NA | adrenal | liver | no | yes | no | no | died | TRM |

| | | | | | | | | | | | |
|---|---|---|---|---|---|---|---|---|---|---|---|
| **M** | 17 | L2 | NA | pelvic | NA | no | yes | no | no | alive | NA |
| **M** | 23 | L1 | NA | adrenal | NA | no | no | yes | no | alive | NA |
| **F** | 18 | Ms | *MYC-N* amplification | adrenal | liver | no | yes | no | no | alive | NA |

**Table S3.** Clinical characteristics of newborns affected by soft tissue sarcoma are summarized. NA = not applicable. PD = progressive disease. TRM = treatment related mortality.

| Sex | Age at diagnosis (days) | Diagnosis | Genomic alterations | Localization | Metastasis | Prenatal diagnosis | Chemo-therapy | Surgery | Relapse | Outcome | Death cause |
|---|---|---|---|---|---|---|---|---|---|---|---|
| **F** | 25 | Congenital Fibrosarcoma | NA | retroperitoneal | no | yes | yes | yes | no | alive | NA |
| **M** | 26 | Embryonal Rhabdomyosarcoma | NA | bladder | no | no | yes | yes | no | alive | NA |
| **F** | 15 | Alveolar Rhabdomyosarcoma | t(2;13) (q35;q14) *PAX3/FKHR* | head/neck | Yes, liver, pancreas, lung, bone marrow, CNS | no | yes | yes | no | died | PD |
| **F** | 22 | Myofibroma | NA | forearm | no | no | no | yes | no | alive | NA |
| **F** | 3 | Malignant rhabdoid tumor | *SMARCA* neg | head/neck | no | no | no | no | no | died | TRM |

**Table S4.** Clinical characteristics of newborns with retinoblastoma are summarized. SCA = segmental chromosomal alterations.

| Sex | Age at Diagnosis (Days) | Familial History | Localization | Metastasis | Prenatal Diagnosis | Chemotherapy | Surgery | Relapse | Outcome | *RB1* Germ Line Mutation |
|---|---|---|---|---|---|---|---|---|---|---|
| M | 11 | yes | unilateral | no | no | yes | no | no | alive | yes |
| M | 20 | yes | bilateral | no | no | yes | no | no | alive | yes |
| M | 0 | yes | unilateral | no | no | yes | no | no | alive | yes |
| F | 15 | no | unilateral | no | no | yes | no | no | alive | no |

**Table S5.** Clinical characteristics of newborns affected by CNS tumors are summarized. NA = not applicable. TRM = treatment related mortality.

| Sex | Age at Diagnosis (Days) | Diagnosis | Metastasis | Prenatal Diagnosis | Chemotherapy | Surgery | Recurrence | Outcome | Death Cause |
|---|---|---|---|---|---|---|---|---|---|
| F | 1 | Ganglioglioma | no | no | no | yes | no | alive | NA |
| F | 5 | Glioblastoma | no | yes | yes | yes | no | alive | NA |
| M | 0 | Choroid plexus carcinoma | no | yes | yes | yes | no | alive | NA |
| F | 0 | Mature teratoma | no | yes | no | no | no | died | TRM |

**Table S6.** Clinical characteristics of newborns affected by other maligniances are summarized. NA = not applicable. TRM = treatment related mortality.

| Sex | Age at Diagnosis (Days) | Disease | Localization | Metastasis | Prenatal Diagnosis | Chemotherapy | Surgery | Relapse | Outcome | Death Cause |
|---|---|---|---|---|---|---|---|---|---|---|
| F | 2 | Mesoblastic nephroma, cellular | Kidney | no | no | no | yes | no | alive | NA |
| M | 6 | Plexiform neurofibroma | Head/neck | no | no | yes | no | no | alive | NA |
| F | 13 | Acute lymphoblastic leukemia, B | Bone marrow | no | no | yes | no | no | alive | NA |
| M | 3 | Akute myeloblastic leukemia | Bone marrow | no | no | yes | no | no | died | TRM |
| F | 1 | Adrenal cortical carcinoma | Adrenal gland | no | yes | no | yes | no | alive | NA |
| F | 9 | Mesoblastic nephroma, classic | Kidney | no | no | no | yes | no | alive | NA |
| F | 1 | Akute myeloblastic leukemia | Bone marrow | no | no | yes | no | no | alive | NA |
| M | 26 | Langerhans cell histiocytosis | Skin of trunk | no | no | no | no | no | alive | NA |
| M | 20 | Langerhans cell histiocytosis | Skin of trunk | no | no | yes | no | no | alive | NA |
